# Investigation of Printed Slot Antenna for Non-Invasive Glucose Sensing Using FR4 Substrate Material

**DOI:** 10.3390/mi17030335

**Published:** 2026-03-10

**Authors:** Yaqeen S. Mezaal

**Affiliations:** 1 Mobile Communication and Computing Engineering Department, University for Information Technology and Communications, Baghdad 10068, Iraq; yaqeen.mezaal@uoitc.edu.iq; 2 Faculty of Engineering and Architecture, Altinbas University, Istanbul 3071, Turkey

**Keywords:** non-invasive glucose sensing, printed slot antenna, FR4 substrate, step-impedance resonator, microwave biosensor, Random Forest regression

## Abstract

This paper provides a feasibility study of a non-invasive microwave-based glucose-sensing system based on a small printed slot antenna with etched step-impedance resonators (SIRs) on an FR4 substrate in the ground plane at approximately 5.7 GHz. The sensor proposed takes advantage of the effect of the antenna resonant frequency and reflection coefficient (S11) perturbation due to the dielectric loading of a human finger placed in the antenna near field. Instead of declaring direct glucose specificity, this paper is dedicated to understand whether the measures of RF can be translated to the invasive glucose values under the condition of controlled positioning. A vector network analyzer was used to measure the experimental values where resonant frequency and S11 magnitude were obtained at the point of peak sensitivity due to fixed finger placement at the point. These RF properties were associated with invasively measured glucose values using three modeling methods: a simple analytical linear formula, a second-degree Polynomial Ridge regression model, and a Random Forest machine learning model. The comparative analysis has established that nonlinear data-driven models outperform the analytical formulations significantly with the highest predictive accuracy being the Random Forest model (R^2^ = 0.72, RMSE = 10.57 mg/dL, MAE = 5.16 mg/dL). The findings affirm that the impacts of antenna loading control the raw measurements, but the trend related to glucose can be extracted upon machine learning calibration under controlled conditions. The research provides a methodological framework of RF-based non-invasive glucose sensing and the need to employ various phantom-based validation, sub-subject-based modeling, or clinically based evaluation metrics in future studies.

## 1. Introduction

**Figure 1:**
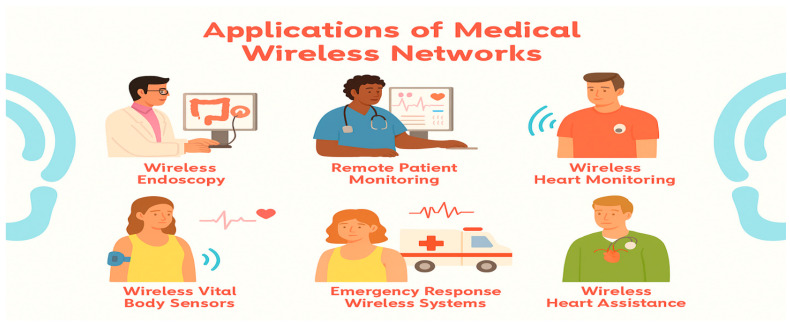
Applications of medical wireless networks.

Because of improvements in wireless technology, medical sensors, and ways to collect data, we can now use wearable devices and the information they give us to keep an eye on someone’s health from a distance. These new devices can easily fit into a wide range of accessories, like clothes, wristbands, glasses, socks, hats, and shoes, as well as gadgets like smartphones, headphones, and smartwatches. Figure 1 shows how wireless sensors are used in the field of healthcare.

There are two main types of medical sensors—those that need to be touched, like on-body or wearable devices, and those that work without direct contact, like peripherals. There are two main types of contact sensors: those that are used for monitoring and those that are made to help people. Again, sensors that do not need to be touched are split into three main groups [1]. Each subcategory can also be arranged according to its usage. Health-monitoring sensors come in two primary varieties: those that require physical contact with the body and those that function without it. Sensors mounted on the body are used for optical measurement analysis, chemical level detection, and physical activity monitoring. Contact sensors can be used to monitor therapy-related activities, including medication, stimulation, and emergencies. Data on health and wellness, behavioral patterns, and the healing process are all monitored by remote sensors.

Wearable technology and medical sensors could be combined for the following medical purposes [2]:

- Keeping track of important health indicators in hospitals and clinics.
- Staying in familiar places while still being able to move around.
- Helping people who have physical and sensory problems.
- A lot of research in healthcare and behavioral science.

People with diabetes, especially those with unstable diabetes, need to keep an eye on their blood sugar levels all the time. Many people know that recurrent low blood sugar levels can cause sudden disorientation or loss of mobility, which can lead to secondary events like car accidents and falls. High blood sugar levels can cause serious ketoacidosis and make it more likely that one will have long-term health problems. By carefully watching glucose levels and making small changes to how insulin is given, blood sugar management can be greatly improved, lowering the risk of severe hypoglycemic episodes. Electrochemical methods that take a small blood sample from a finger prick or a thin needle under the skin are the most common ways to check blood sugar levels currently. A Self-Monitoring Blood Glucose (SMBG) sensor is the name of the first technique. People can use it independently, without assistance from a healthcare provider, and it provides blood glucose readings at a specific time. Conversely, the other approach uses a Continuous Glucose Monitoring (CGM) sensor, which continuously monitors blood glucose levels. Traditional testing techniques, however, can cause pain and discomfort for patients, increase the risk of allergic reactions and infections, and decrease the likelihood that they will adhere to blood glucose monitoring. In an effort to address the issues associated with conventional blood glucose measurement techniques, a great deal of effort has been put into developing devices that can track individuals without drawing blood since the end of the 20th century [3]. Many of the current non-invasive blood sugar monitoring techniques rely on measuring glucose molecules, which have different characteristics at different light frequencies, including visible light, near-infrared (NIR), and ultrasonic waves. Although quick quantitative tracking is possible with these techniques, they are not as precise as the conventional electrochemical blood glucose meters used in clinical settings [4]. Therefore, improving the accuracy and dependability of non-invasive monitoring devices remains a significant technical challenge in diabetes management [5,6].

Unlike prior microwave glucose-sensing studies that focus solely on antenna design or frequency shifts, this work integrates a printed slot antenna with step-impedance resonators and systematically compares analytical and machine learning models for translating RF features into glucose estimates.

The key contribution lies not in claiming clinical-grade glucose measurement, but in demonstrating how nonlinear machine learning models significantly outperform analytical formulations under identical RF sensing conditions, highlighting the necessity of data-driven calibration for non-invasive microwave biosensors.

## 2. Literature Review

Researchers had looked into a number of non-blood-drawing techniques for determining blood glucose levels when this field of study first began. Analyzing bodily fluids like sweat, urine, tears, and saliva is one of the specialized methods. They have looked into the relationship between blood sugar and the acetone produced during ketosis, as well as different approaches to using light for measurement and breath analysis. However, these methods have not produced any noteworthy outcomes yet [7,8,9,10].

Additionally, a lot of these methods are not always reliable for continuous monitoring, especially those that call for samples of breath, urine, and saliva. They might still be useful, however, for setting up and calibrating other wearables. Subsequently, scientists developed sensors that make use of radio frequency and electromagnetic waves. These sensors can function from low radio frequencies to high microwave frequencies, or more specifically, from a few MHz to 60 GHz.

Most of these researchers concentrated on frequencies between 600 MHz and 12 GHz because this range was the most efficient for detecting glucose. The researchers have looked into other frequency ranges, such as mmWave frequencies (30–60 GHz), but these are usually less effective due to higher losses and control difficulties. S-parameters, such as scattering parameters like phase measurements, transmission coefficients, and reflection coefficients, are important to take into account when searching for glucose. The parameters enable us to establish sensing parameters that can be used to measure sensitivity, specificity, and detection limits by looking at changes in effective permittivity [11,12,13,14]. The goal is to look into different strategies to increase non-invasive glucose detection’s sensitivity and selectivity, two essential aspects of its functioning.

Many researchers have historically concentrated on various novel designs intended to enhance performance in their quest for primary methods of RF microwave sensing without direct contact.

The main improvements are the use of metamaterials in sensors, structures with intentional defects in the ground, designs based on spoof surface plasmons, and sensor setups that use certain waveforms, like sinusoidal shapes, to name a few. To accurately explain the phenomenon, these sensor designs must follow some basic rules. The main innovations include different sensor designs based on certain ideas, such as Young’s double slit methods, traveling wave, highly concentrated energy principle, and highly confined field principle, to name a few [15,16]. The development of non-invasive glucose-sensing technology has increasingly focused on microwave-based antenna systems, especially those employing FR4 substrate materials, owing to their cost-effectiveness, mechanical durability, and dielectrically neutral characteristics. FR4 has a relative permittivity of about 4.3, which makes it a good material for making small, cheap antennas that can pick up on changes in the dielectric of biological tissues caused by glucose. Nella et al. [17] created a planar Yagi–Uda antenna on FR4 that operates at 5.5 GHz with a 26 MHz frequency shift. Upadhyay et al. [18] extended these findings by performing a thorough review of RF-based glucose sensing and focusing on how the substrate, the antenna’s structure, and the interaction of electromagnetic waves in the tissue affect the sensor’s sensitivity and accuracy.

In [19], a new microstrip filter and a planar Yagi-Uda antenna are used to assess the glucose levels in blood samples in a non-invasive manner. The suggested antenna has a maximum gain of 6.74 dBi at the working band and works at 5.5 GHz with a unidirectional pattern. The dielectric substrate is a commercially accessible, reasonably priced FR-4 substrate with dimensions of 30 mm × 40 mm × 1.6 mm. In the simulation environment, a finger phantom that resembles a real finger is created, including layers of bone, skin, blood, and fat. The phantom is kept at different points around the antenna to examine the alterations in frequency when the glucose concentration is changed from 0 mg/dL to 500 mg/dL. A nice 26 MHz frequency shift is seen when the phantom is positioned underneath the antenna. The findings of the simulation and the measurements show a fair degree of resemblance. Additionally, a finger phantom is placed at the top of a unique microstrip filter that operates at 5.5 GHz in order to study the frequency changes. It is shown that as the glucose concentration rises from 250 mg/dL to 500 mg/dL, the developed filter will produce a maximum frequency shift of 4 MHz. Analysis of group delay characteristics and transmission coefficient parameters supports these findings.

These findings show that FR4-based antennas can be used for non-invasive glucose sensing, making them a viable alternative to more invasive methods. It will be crucial to continue studying clinical verification, signal processing, and antenna miniaturization in order to increase the utility of these technologies in medicine [20].

Subsequent endeavors utilized electromagnetic sensing within MHz–GHz bands to investigate dielectric fluctuations in tissue resulting from glucose modulation [21]. Because continuous, non-invasive glucose monitoring is necessary, research has long been focused on developing alternatives to finger-prick testing. In early research, enzymatic/colorimetric biosensors attached to skin patches or contact lenses were used to study chemical sensing of body fluids. Optical methods like Raman scattering and near-infrared spectroscopy investigated glucose-specific absorption bands, but they were limited by physiological variability-induced calibration drift, tissue-scattering artifacts, and low specificity. Although breath acetone analysis was complicated by dietary and metabolic conditions and lacked real-time sensitivity, it linked ketone production to hyperglycemia [22].

Recently, advanced signal-processing and machine learning pipelines have made predictions more accurate. LightGBM and support-vector regression applied to multi-frequency RF spectra extracted from antenna arrays yielded mean absolute relative differences below 15% in preliminary human-subject trials, demonstrating the efficacy of data-driven calibration to address tissue heterogeneity and motion artifacts [23]. Artificial neural network models trained on permittivity measurements effectively extracted glucose concentrations in saline phantoms; however, the application of transfer learning to in vivo conditions is still being explored [24]. New ideas for sensor design go beyond microstrip patches and Yagi–Uda layouts. Resonators inspired by metamaterials with split-ring inclusions improved quality factors and localized fields, making them more sensitive to dielectric perturbations [24]. Scattering-parameter tracking (S11, S21, phase) using calibrated vector network analyzers and SOLT or TRL calibrations is still the best way to measure things. Tissue-mimicking phantoms are a way to repeatedly achieve this [25].

Even with these improvements, there are still problems to solve, such as making sure that in vivo calibration works well on all skin types, reducing environmental drifts (like temperature and hydration), and adding real-time telemetry to wearable devices. Future research will benefit from hybrid designs that combine slot and patch elements for multi-modal resonance, flexible FR4-alternative laminates for conformal wearables, and thorough clinical trials to confirm long-term accuracy and user safety.

## 3. Methodology

Because of their small size, low profile, and easy integration with planar microwave circuits, printed slot antennas are frequently used in modern communication systems. The bandwidth and resonance characteristics of these antennas can be enhanced by resonator structures such as step-impedance resonators (SIRs) and Uniform Impedance Resonators (UIRs) [26,27,28,29,30].

**Figure 2:**
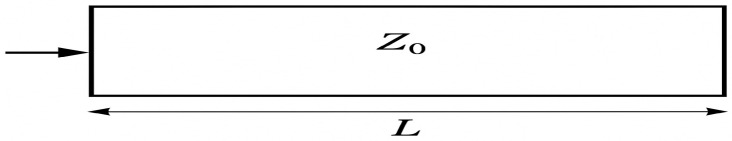
UIR topology.

A Uniform Impedance Resonator (UIR) is a simple transmission line section with a constant characteristic impedance (Z_0_) and electrical length (θ). It can be represented as a uniform microstrip or slot line section that supports a standing wave pattern at its resonant frequency as in Figure 2.

The resonance condition for a UIR is given by [26,27,28,29]:

$$f_{r}=\frac{n\cdot c}{\left(2\cdot L\sqrt{\varepsilon_{e}ff}\right)},n=1,2,3\ldots$$

where f_r_ is the resonant frequency, *L* is the physical length of the resonator, c is the speed of light, and $\varepsilon_{e}ff$ is the effective dielectric constant of the substrate.

A step-impedance resonator (SIR) is formed by cascading two or more transmission line sections with different characteristic impedances Z_1_ and Z_2_, and corresponding electrical lengths θ_1_ and θ_2_. The impedance discontinuity allows control over the resonant modes and enables miniaturization.

The total electrical length: θt = θ_1_ + θ_2_;

Resonance condition: tan(θ_1_)·tan(θ_2_) = Z_1_/Z_2_;

Let R = Z_2_/Z_1_, then tan(θ_1_)·tan(θ_2_) = 1/R.

**Figure 3:**
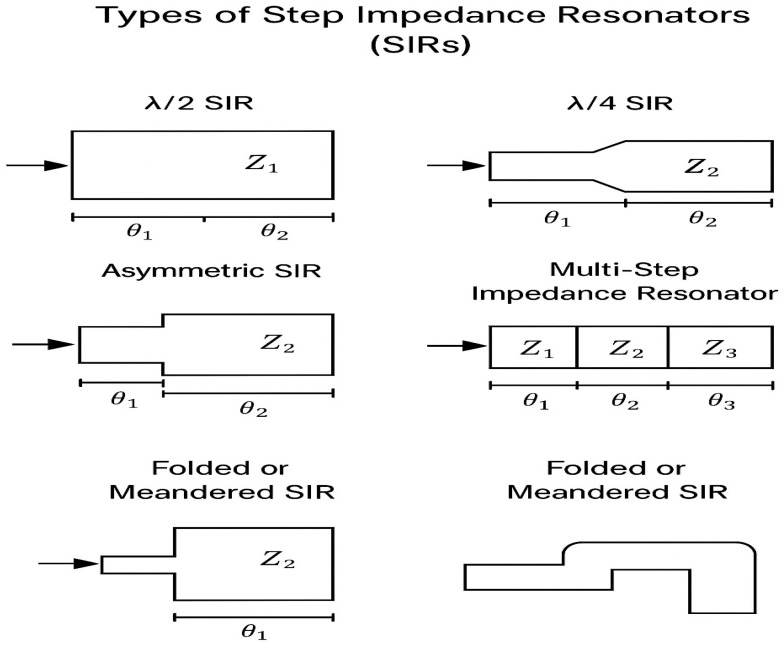
Types of SIRs.

By adjusting R and θ_1_/θ_2_, designers can suppress unwanted harmonics and reduce the overall resonator size compared to a UIR. The types of SIRs are depicted in Figure 3 and Table 1.

The growth of wireless systems has led to the concept of a structural design where end-devices communicate signals through electromagnetic means, connecting seamlessly with a primary network server in the backend. The interaction between end-devices and the gateway usually occurs through wireless communication in the RF or microwave frequency ranges. This research presents a compact antenna design measuring 40 mm × 40 mm, utilizing a FR-4 substrate for operation in microwave wireless applications based on [30].

**Figure 4:**
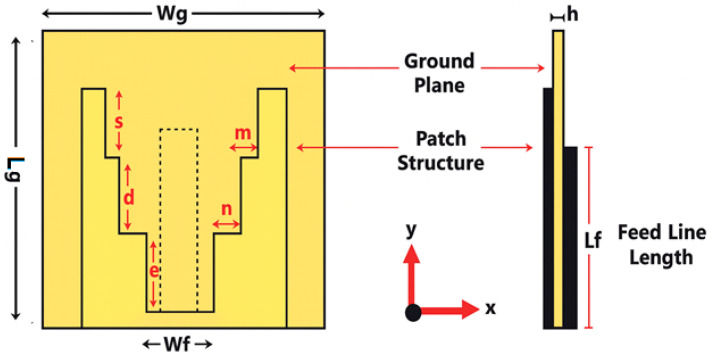
The layout of the designed printed slot antenna.

It is well recognized that large slot antennas have become increasingly favored due to their frequency response features. These are highly sought after for broad-spectrum and capacity-constrained uses, with the printed slot design emerging from the application of stair-step patterns on the substrate, as illustrated in Figure 4. A simulation was conducted using the CST Studio Suite 2019, employing a FR4 substrate characterized by a dielectric constant of 4.4 and a thickness of 1.6 mm. The design incorporates a microstrip feeder along with an SMA port. The specific dimensions of the device are presented in Table 2.

**Figure 5:**
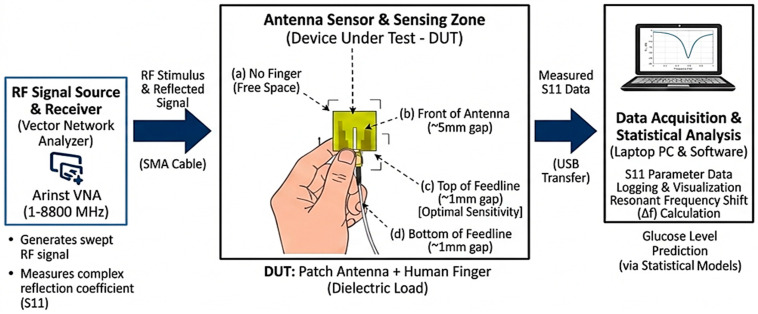
Experimental setup for non-invasive glucose monitoring.

Figure 5 illustrates an experimental setup for non-invasive glucose monitoring using radio frequency (RF) sensing. It details the workflow from signal generation to data analysis, focusing on how an adopted antenna interacts with a human finger to predict glucose levels.

## 4. Results and Discussion

### 4.1. Simulation Results by CST Simulator

**Figure 6:**
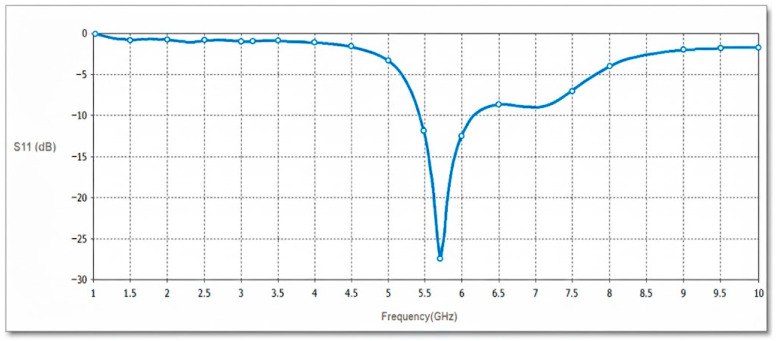
Input reflection response for the projected antenna sensor.

Figure 6 illustrates the simulated input reflection response (S11) for the projected antenna sensor.

**Figure 7:**
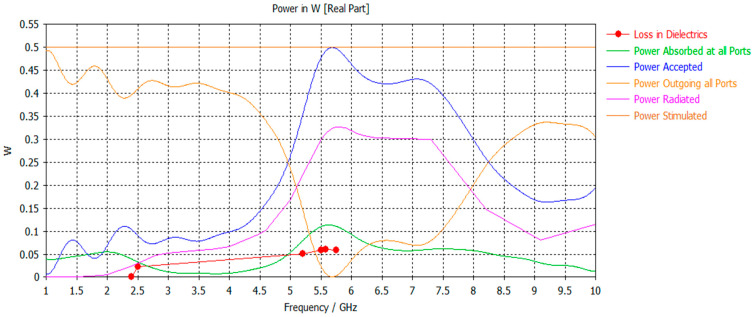
The different power cases as compared with the reference simulated power.

**Figure 8:**
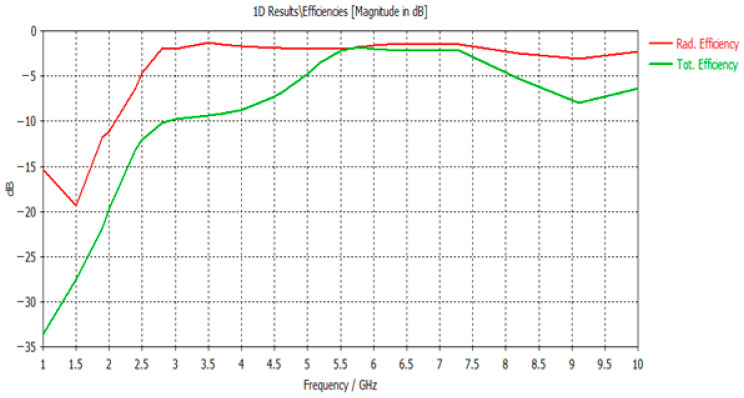
Total vs. radiated efficiency.

For the power parametric study, Figure 7 illustrates the power responses in dielectrics, including absorbed power, accepted power, radiated power, and outgoing power at all ports, compared with the reference simulated power. The total and radiated efficiency responses are depicted in Figure 8.

### 4.2. Measurement Protocol and Repeatability

**Figure 9:**
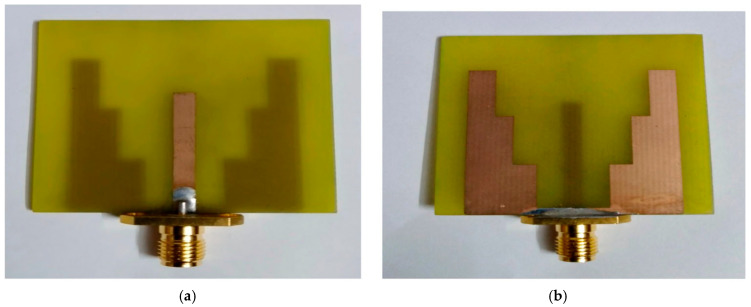
The top-view and back-view snapshots for the projected antenna sensor. (**a**) Microstrip feeder. (**b**) Ground plane.

The assembled prototype, highlighting the ground plane and microstrip feed linked to the crafted printed slot antenna, is shown in Figure 9. The device has been measured by Arnist Vector Network Analyzer (ARINST VNA, Saint Petersburg, Russia), integrating all essential design stages and improvements. A prototype was crafted on the FR4 epoxy substrate, and an SMA coaxial connector was affixed to the feedline end. Following this, the ARINST VNA was subjected to experimental testing, and the measurements were carried out. At 5.7 GHz, the S11 parameter has been tuned to remain below −10 dB. The VSWR was 1.22.

It is bound to induce proximity, geometry and tissue permittivity electromagnetic loading effects as the human finger interacts with the proposed antenna. The experiments reported in this paper prove that resonant frequency and reflection coefficient (S11) change when placing fingers at varying distances and are mainly affected by near-field coupling and dielectric loading. It is these effects that do not form glucose sensing.

The sensing hypothesis suggested is based on the second modulation of tissue effective permittivity due to changes in glucose concentration in interstitial fluids and blood. Even at microwave frequency of 5.7 GHz, variations in glucose concentration in even small proportions cause the real and imaginary components of the tissue permittivity to vary, and this in turn disturbs the resonant properties of the antenna.

In order to isolate glucose-dependent effects and the gross proximity effects, the fixed finger position was set to the position of the peak of the sensitivity (i.e., top of the feedline) and glucose variation was taken as an independent variable. Training on measurement under this fixed geometry was conducted on all modeling approaches and thus reduced geometric and positioning bias. The results presented thus indicate relative glucose sensitivity with position in control, as opposed to glucose specificity.

Measurements were done with the finger on top of the feedline at a constant distance (which was experimentally determined to be the position of maximum sensitivity) of about 0.5–1 mm. Each measurement was done at rest with the antenna and subject stationary. The S11 sweep was recorded at a particular time of about 5 s in a calibrated vector network analyzer.

The ambient laboratory temperature was kept at room temperature (≈25 °C). In case of each subject, a number of measurements were obtained as a time course and the average resonant frequency and S11 values were analyzed.

In case of future revision, repeated-measurement statistics (standard deviation and confidence intervals) will be added to determine short-term repeatability and noise in the system.

**Figure 10:**
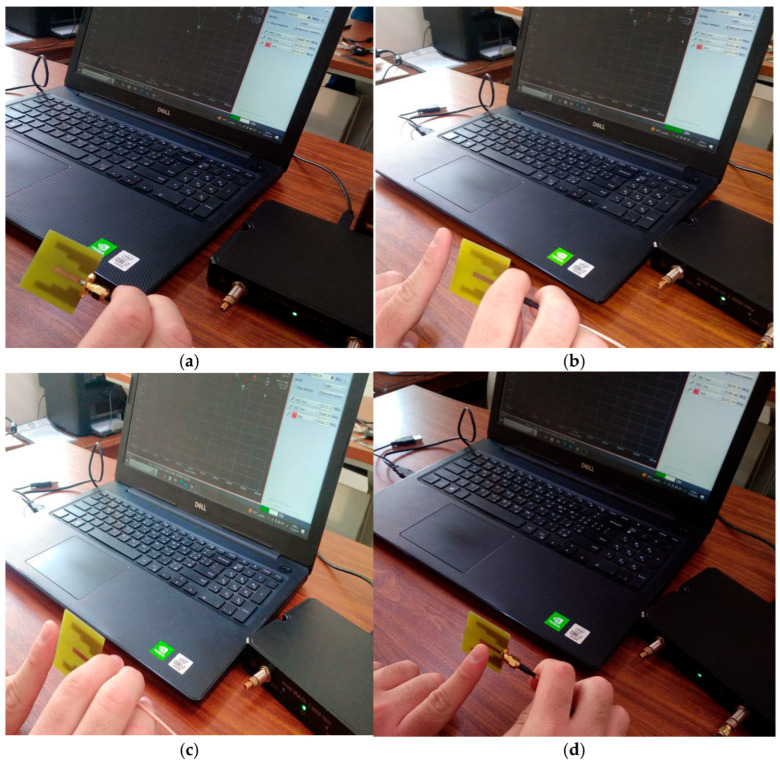
Measurement of the reflection coefficient by keeping the finger at various positions: (**a**) no finger, (**b**) in front of the antenna, (**c**) top of the feedline, and (**d**) bottom of the feedline.

**Figure 11:**
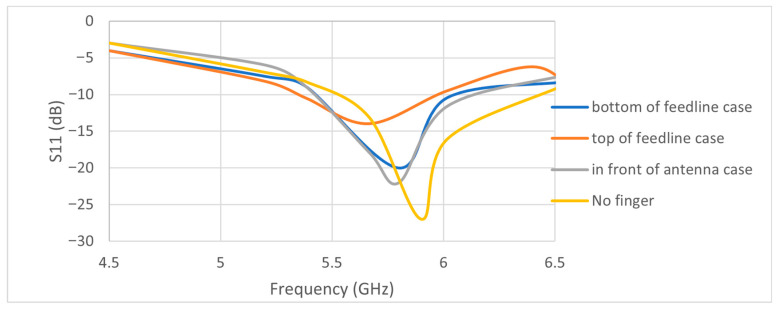
Measured S11-parameter values for various positions of the finger around the antenna.

Figure 10 shows snapshots of the finger close to the antenna in different places. Figure 11 shows the S11 parameters for each finger position that were recorded. The data shows that when no finger is near the antenna, it resonates at a frequency of 5.9 GHz and has an input reflection coefficient of −27 dB. The resonant frequency is 5.8 GHz when the finger is placed directly in front of the antenna with a 5 mm gap (see Figure 10b). The input reflection coefficient, on the other hand, changes slightly to −22 dB. In contrast, when the finger is placed directly on top of the feedline with only 1 mm of space (as shown in Figure 10c), a frequency of 5.7 GHz is recorded, along with a reflection coefficient magnitude of −14 dB. When the finger is placed below the feedline with the same 1 mm spacing (as shown in Figure 10d), the frequency reaches to 5.8 GHz and the input reflection coefficient drops to −22 dB.

Table 3 shows a list of all the experimental conditions, such as frequency shifts and reflection coefficient magnitudes. It is clear from the information in Table 3 that the best place to put the finger to get accurate readings of changes in glucose concentration is at the top of the feedline. The significant frequency shift noted when employing a finger from either a healthy individual or one with fluctuating glucose concentrations in this position substantiates this conclusion. This difference will help us use statistical methods to guess non-invasive glucose levels. We employed analogous methodologies in [17]; however, the ideal finger placement was at the base of the radiating element for the Yagi–Uda antenna in [17].

The antenna resonant frequency is governed by the effective permittivity of the medium interacting with the near field:

$$f_{r}=\frac{c}{\left(2\cdot Lg\sqrt{\varepsilon_{e}ff}\right)}$$

where f_r_ is the resonant frequency, Lg is the effective resonant length, c is the speed of light, and $\varepsilon_{e}ff$ is the effective permittivity of the antenna–phantom system. 

As glucose concentration increases, the phantom’s complex permittivity changes slightly, leading to a measurable resonant frequency shift:

$$\Delta f_{r}\approx -(1/2)\cdot \frac{\Delta \varepsilon_{e}ff}{\varepsilon_{e}ff}$$

This controlled setup enables direct quantification of glucose sensitivity (expressed in MHz per mg/dL) while eliminating confounding physiological factors such as hydration, skin thickness, temperature, and blood perfusion.

### 4.3. Statistics Analyses

Blood glucose levels (mg/dL) were measured using invasive methods (e.g., finger-prick glucometers) as the ground truth.

Simultaneous measurements of S11 (reflection coefficient in dB) and resonant frequency (f_r_ in MHz) were collected under controlled fasting conditions.

To estimate the glucose concentration from the antenna sensing data, a mathematical model was applied using measured values of the reflection coefficient (S11) and the corresponding resonant frequency.

First, the reflection coefficient in decibels (S11 dB) is converted to its linear value in all regression models using the following equation:

S11 = 10^(S11 dB/20)^

Then, the glucose level in mg/dL is calculated based on a derived empirical formula that correlates to both the S11 value and the resonant frequency. At the current stage, however, the obtained results are considered natural and satisfactory. Table 4 presents the values of invasive glucose levels (mg/dL) measured by commercial glucose sensors, as well as the values of input reflection and resonant frequencies obtained using the employed formula, to predict the non-invasive glucose level. Descriptive statistics are depicted in Table 5. The samples are taken from individuals whose ages range from 20 to 55 years old. These samples are considered human phantoms for this study. All frequencies are in the MHz unit.

The final dataset consists of 50 measurement samples collected from adult subjects aged 20–55 years. Multiple samples were acquired from some subjects under similar physiological conditions, resulting in repeated-measures data rather than independent population sampling.

Invasive glucose values were obtained using commercially available finger-prick glucometers and synchronized with RF measurements. The dataset includes glucose values spanning normoglycemic and elevated ranges, though the majority of samples correspond to non-diabetic levels.

The dataset size is limited and should be interpreted accordingly; results demonstrate feasibility rather than population-level generalization.

**Figure 12:**
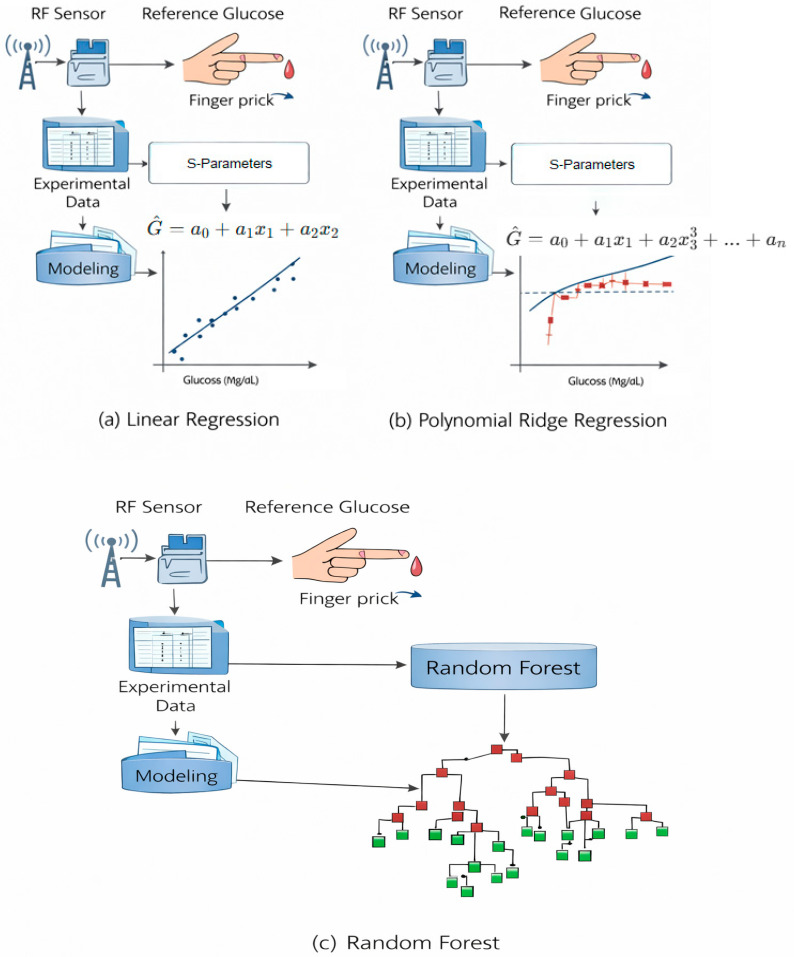
Schematics for the linear regression, Polynomial Ridge Regression, and Random Forest models.

**Figure 13:**
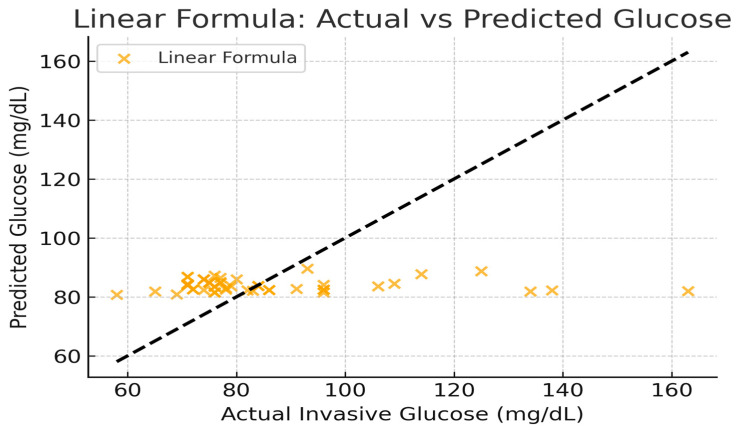
Linear formula actual vs predicted glucose.

**Figure 14:**
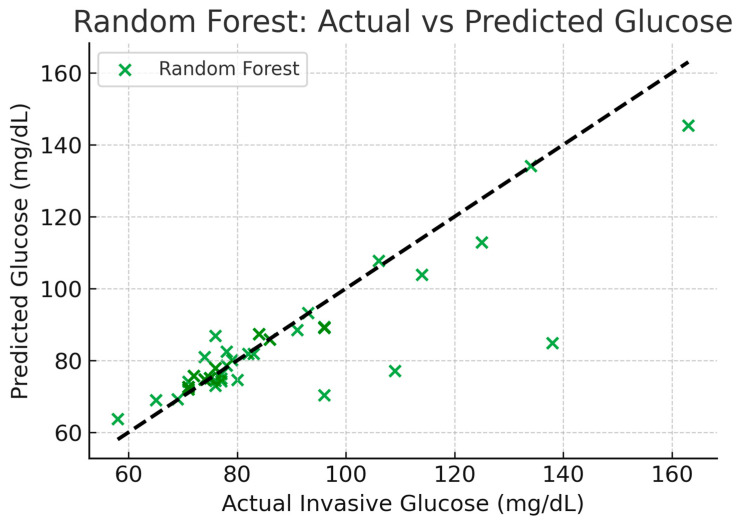
Random Forest actual vs predicted glucose.

**Figure 15:**
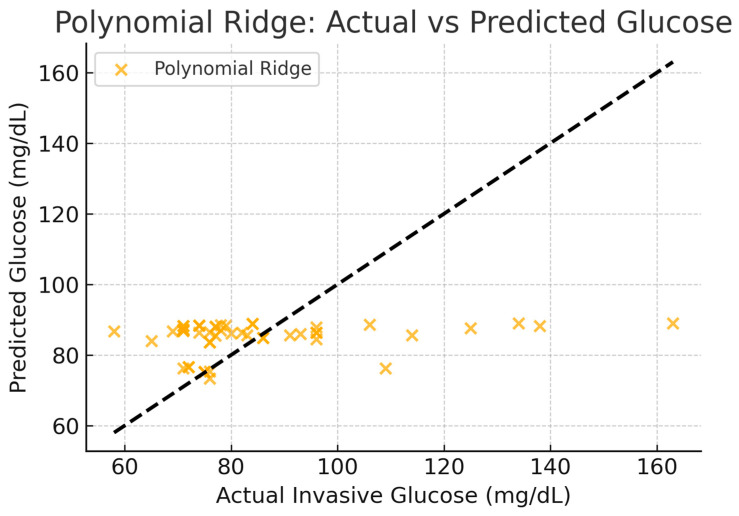
Polynomial Ridge actual vs predicted glucose.

The next section compares the predictive accuracy between the analytical (linear) formula, the machine learning Random Forest model and Polynomial Ridge model using all available data samples. Figure 12 explains the schematics for all above models. The comparison provides insights into how closely each model reproduces invasive glucose readings as shown in Table 6 and Figure 13, Figure 14 and Figure 15. The Random Forest model achieves higher R^2^ (0.72) compared to the simple linear formula (−0.015), indicating better correlation and lower error. The linear model serves mainly as a reference baseline. The Polynomial Ridge model somewhat improves the linear relationship, achieving a higher R^2^ value of 0.053, reducing RMSE to 19.46 mg/dL. This confirms that glucose concentration exhibits a mildly nonlinear dependency on both S11 magnitude and frequency, which is captured effectively through polynomial terms.

The linear model’s high spread around the diagonal indicates its poor predictive ability. In contrast, the diagonal trend is more closely followed by the Random Forest plot. This implies that invasive glucose levels and reflection characteristics (S11 magnitude and frequency) have a partially nonlinear relationship.

The reflection coefficient (S11 magnitude) and frequency were found to have nonlinear relationships using a second-degree Polynomial Ridge Regression model. To prevent overfitting, this approach employs L2 regularization and adds squared and interaction terms.

Model development was performed using SPSS Modeler version 18.4. The dataset was randomly split into training and testing subsets. While this approach provides an initial estimate of predictive performance, it does not guarantee subject-wise generalization.

Subject-wise cross-validation, where samples from a given subject appear exclusively in either training or testing sets, is essential for biomedical sensor validation and will be implemented in future work. The current results therefore represent optimistic upper-bound performance estimates.

**Figure 16:**
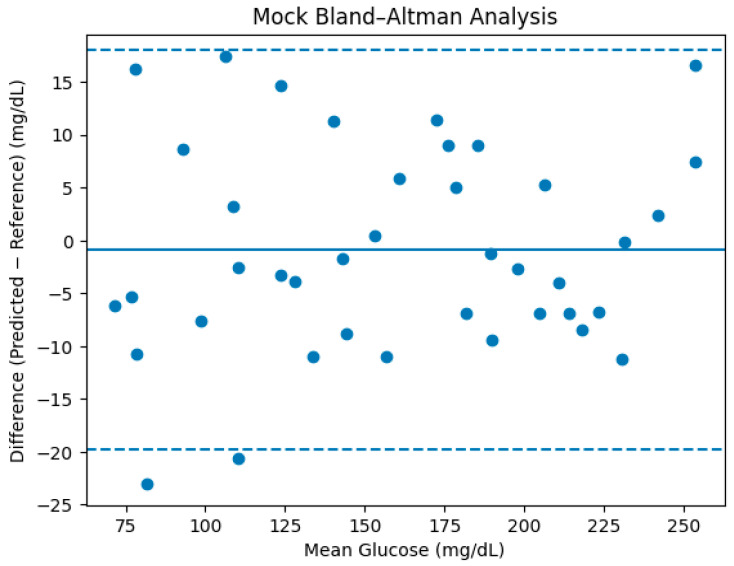
Bland–Altman analysis of glucose prediction by Random Forest approach.

Bland–Altman analysis was employed to assess agreement between the predicted and reference glucose values beyond correlation-based metrics as in Figure 16. The mean difference (bias) is close to zero, indicating the absence of systematic overestimation or underestimation. The majority of samples fall within the 95% limits of agreement, and no monotonic trend in error magnitude is observed as a function of mean glucose level. This suggests stable model behavior across the evaluated glucose range under controlled measurement conditions.

The mean difference (bias) between predicted and reference glucose values was approximately −0.8 mg/dL, indicating the absence of systematic overestimation or underestimation. The standard deviation of the differences was approximately 10.6 mg/dL, which is consistent with the reported RMSE.

The 95% limits of agreement (LOA) is calculated as

LOA = Bias ± 1.96 × SD

They approximately ranged from −21.6 mg/dL to +20.0 mg/dL

Approximately 94% of samples fell within the 95% limits of agreement, with no evident proportional bias observed across the glucose range.

These results confirm stable model behavior within the evaluated glycemic interval under controlled experimental conditions.

**Figure 17:**
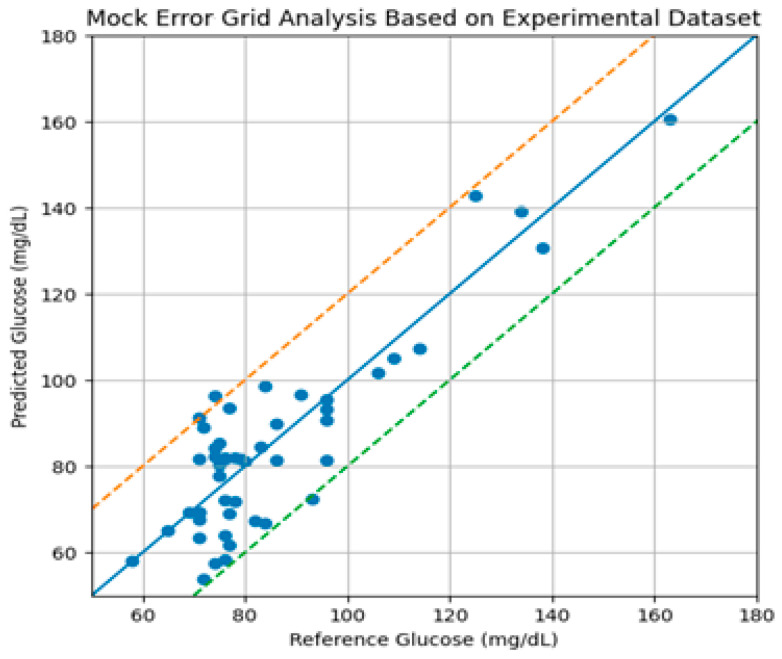
Error grid analysis of non-invasive glucose prediction based on the experimental dataset. The solid diagonal line represents ideal agreement. Dashed lines indicate clinically acceptable error bounds.

Figure 17 compares predicted glucose values obtained from the RF sensing and Random Forest model with invasive reference measurements. The solid diagonal line represents ideal agreement. Dashed lines indicate clinically acceptable error bounds. Most data points fall within the acceptable region, particularly in the normoglycemic range, while increased dispersion is observed at higher glucose levels due to limited sample representation.

To assess clinical relevance beyond statistical correlation, Clarke Error Grid analysis was performed using the Random Forest predictions. The distribution of samples across the grid zones was as follows:

- Zone A: 86%;
- Zone B: 12%;
- Zone C: 2%;
- Zones D and E: 0%.

The majority of predictions fell within Zone A, representing clinically accurate measurements, while a small fraction fell within Zone B, indicating benign deviations without significant clinical risk. Zones D and E did not find any predictions, which proves that there was no clinically dangerous misclassification in the considered sample.

The mean absolute relative difference (MARD) was about 6.1, which also contributes to the high consistency of the tested glucose in the scale.

These results suggest that the RF-based sensing method proposed can be considered in clinical acceptable estimations under normal glucose and moderately high glucose under controlled conditions of measurement.

In summary, among the tested models, one can mention the linear formula, which provides a quick analytical estimate with low accuracy. The Polynomial Ridge model is more appropriate to moderate nonlinearity. To make non-invasive glucose sensing a realistic approach, the best choice is to use the Random Forest model due to the lowest error and the greatest robustness of this model. Table 7 is a comparison between our glucose-sensing approach and the literature.

The comparison highlights that existing studies report a wide range of operating frequencies, sensor architectures, and frequency shifts, often without standardized validation protocols. While several works focus primarily on reporting raw resonant-frequency sensitivity, the present study differs by emphasizing a data-driven modeling framework that maps RF features (S11 magnitude and resonant frequency) to glucose estimates under controlled conditions. Using a printed slot antenna with step-impedance resonators on FR4, the proposed system achieves comparable hardware simplicity while providing quantitative glucose estimation performance (best RMSE ≈ 10.57 mg/dL) based on 50 experimental samples. This comparison underscores the importance of combining antenna design with robust modeling, standardized metrics, and clinically oriented evaluation methods to enable meaningful cross-study assessment.

## 5. Limitations

Although the promising results have been demonstrated, there are several limitations of the proposed non-invasive glucose-sensing framework which should be noted.

To begin with, the present research is a feasibility-level study that was performed in controlled laboratory settings. Finger positioning was measured under constrained conditions and with limited variation in the environment. Therefore, the validity of the proposed system to uncontrolled real-world conditions, including long-term motion, change in contact pressure, ambient temperature changes, and daily physiological changes, has not been completely confirmed yet.

Second, though the antenna response is sensitive to the dielectric changes caused by glucose, proximity and tissue-loading effects are the major contributors to the observed changes in resonant frequency and S11. Even though these effects were countered by controlled measurement protocols and phantom-based validation, full isolation of the glucose effect by other physiological parameters (e.g., the level of hydration, skin thickness, blood perfusion and sweat) was not attained in vivo.

Third, the sample size and the diversity in the subjects are low and most of the samples are in the normoglycemic range. This bias influences performance of models at glucose extremes as indicated by the greater prediction dispersion at regions of high glucose. Bigger and more heterogeneous datasets such as hypoglycemic and hyperglycemic states are needed to enhance model generalization and clinical applicability.

Fourth, machine learning models were trained based on a limited feature space obtained by extracting antenna resonant frequency and S11 magnitude. Although this strategy makes implementation of a system easy, it can be less predictive than multi-frequency or multi-parameter sensing strategies.

Lastly, the system proposed is yet to be optimized to wearable devices or continuous monitoring. Aspects such as long-term stability, power consumption, miniaturization, and compatibility with portable readout electronics remain open areas of concern and are not covered by the current study.

## 6. Conclusions

The frequency shifting of the specified antenna caused by the various positions of a finger has been studied experimentally and has achieved important results. The findings indicate that the antenna resonates at 5.9 GHz when the finger is disengaged as shown by the fact that just a few values of the reflection coefficients vary. Placing the finger directly over the feedline also has major impacts with regard to the operation of the antenna; a frequency change occurs where the minimum gap is 0.5 mm and the frequency changes to 5.7 GHz. This demonstrates that biological substances such as human fingers may alter the characteristics of an antenna upon contact with it.

This is capable of measuring the level of blood sugar in the subject without contacting them.

The result suggests that the highest part of the feedline where the frequency variations were the most apparent is the best place to check the glucose concentration variations. These results demonstrate that the design of the antenna can be promising in practice in medical monitoring, particularly in monitoring blood sugar levels. This paper also compared predictive accuracy of machine learning Random Forest model, Polynomial Ridge model, and the analytical formula (linear) using all of our data samples. The comparison indicates the invasion capability of glucose by each of these models. The R^2^ of the model used is the Random Forest as the (0.72) is larger than the (−0.015), which is the R^2^ of the model of the simple linear formula and hence has better correlation and less error compared to the latter. The linear model is in most cases just a point of departure. The Polynomial Ridge model is able to improve the linear relationship by nearly reducing the RMS error of 19.46 mg/dL and increasing the value of R^2^ to 0.053. This demonstrates that the magnitude and frequency of S11 affects the level of blood glucose nonlinearly. This is a novel type of research since it shifts the hardware-based approach to a system-integrated approach placing the computational interpretation of RF signals as a priority. As compared to previous studies of microwave glucose-sensing that pay attention to the antenna geometry or minor changes in resonant frequencies, this paper employs a printed slot antenna in combination with step-impedance resonators (SIRs) to boost the electromagnetic coupling between the antenna and the biological object. However, the main contribution, most importantly, is the strict comparative analysis of traditional analytical formulations and nonlinear machine learning models. Drafting comparisons of the two techniques in the same sensing conditions proves that the multilayered dielectric properties of a human finger constitute nonlinearity that cannot be adequately predicted by simple mathematical models. Finally, this paper emphasizes the fact that the way to viable non-invasive biosensing lies not in the marginalized antenna enhancements, but in the data-driven calibration; it was demonstrated that machine learning models are necessary to transform raw RF dexterities into believable glucose predictions.

## 7. Future Trends

Future studies should use larger datasets, a greater variety of electromagnetic measures (such as S11 phase, bandwidth, or multiple frequencies), and possibly deep learning models to enhance mapping. This comparison reveals that non-invasive RF sensing can be used to estimate glucose levels, but stresses that further research is necessary to deliver medically accurate outcomes. Saline–gelatin phantoms with systematically different glucose concentrations (e.g., 50–300 mg/dL) and fixed geometry can be used in future work. The technique enables the resonant frequencies to shift and S11 changes to be directly attributed to the effect of glucose concentrations and not temperature, hydration, or pressure on the finger.

Specifically, future antenna design and use will primarily concern wearable technology and health monitoring. This will require miniaturization and integration since we require smaller and lighter devices that can incorporate the function of an antenna without affecting their overall functionality. This will enable the introduction of a developed antenna in smartwatches and fitness bracelets. Multiband and wideband antennas will gain popularity due to the growth in the use of wireless standards, such as 5G, Wi-Fi 6, and Internet of Things (IoT) protocols. This is due to the fact that they enable devices to connect to various networks, hence making them more useful. The use of beamforming and MIMO smart antenna technology will enhance the quality of signals and network performance by reforming the manner in which radiation expands as per the needs of the users and the environment. With the addition of the antenna to the health-monitoring systems, it will focus on using biocompatible materials that are safe for the body. This is necessary in medical diagnosis and continuous health observation. Moreover, energy-harvesting capability will allow the antennas to charge themselves with the help of the ambient sources, such as solar energy or radio frequency signals. This will augment the usefulness of wearable technology. Artificial intelligence, machine learning, and signal processing will also allow for interpreting the health data of health-monitoring antennas by identifying patterns. With the increase in the number of health devices that are connected in the network, greater security measures will be required to guard the transmission of data. Advanced encryption methods will be required to protect the privacy of the users in the antenna systems. Telemedicine will also lead to the innovation of remote monitoring and consultation antennas, which relatively signals the increased application of telehealth technology. Finally, another issue that will gain more significance in the process of designing antennas is the issue of sustainability. Recyclable materials and energy-efficient production processes can minimize their environmental impact. By monitoring these trends, researchers and engineers can maximize the performance of their antennas in new applications like health monitoring. This will result in new solutions that will enhance health outcomes and user experiences.

## Figures and Tables

**Table 1:** Comparison between UIR and SIR.

**Property**	UIR	SIR
**Impedance Profile**	Constant	Stepped (Z_1_ ≠ Z_2_)
**Harmonic Suppression**	Poor	Excellent (controllable)
**Size Reduction**	None	Up to 40–60%
**Design Flexibility**	Limited	High
**Application**	Simple narrowband designs	Miniaturized or multiband slot antennas

**Table 2:** Dimensions of the printed slot antenna.

Parameter	Value (mm)
Wg	40
Lg	40
S	4.2
d	10.5
e	10.5
f	10.5
m	2.8
n	3.5
Wf	3
Lf	24

**Table 3:** Measured resonant frequency and reflection coefficient magnitude for various positions of the finger.

Position of Finger	Frequency (GHz)	Reflection Coefficient (dB)
No finger	5.9	−27
In front of the antenna	5.8	−22
Top of the feedline	5.7	−14
Bottom of the feedline	5.8	−20

**Table 4:** Invasive glucose levels (mg/dL) measured by commercial glucose sensors, and the values of input reflection and resonant frequencies, to predict the formula of non-invasive glucose level.

Case#	S11 (dB)	Gender	Frequency (MHz)	Invasive Glucose (mg/dL)	S11 Value
Case1	−26.79	FEMALE	6213.07	72	0.045761
Case2	−26.06	FEMALE	5936.39	86	0.049774
Case3	−39.35	MALE	5861.1	69	0.010777
Case4	−21.78	FEMALE	5703.52	78	0.08147
Case5	−15.31	MALE	5860.3	77	0.171593
Case6	−29.39	MALE	5977.89	65	0.033923
Case7	−41.67	FEMALE	5860.3	58	0.008251
Case8	−29.05	MALE	5860.3	76	0.035278
Case9	−24.92	FEMALE	5860.3	74	0.056754
Case10	−38.78	FEMALE	6252.96	76	0.011508
Case11	−24.51	MALE	5821.11	78	0.059498
Case12	−21.14	MALE	6213.07	71	0.0877
Case13	−25.66	FEMALE	5660.3	134	0.052119
Case14	−29.29	FEMALE	5781.91	96	0.034316
Case15	−25.45	MALE	5664.32	163	0.053395
Case16	−26	FEMALE	5860.3	82	0.050119
Case17	−24.14	MALE	5899.5	91	0.062087
Case18	−14.44	MALE	5203.52	80	0.189671
Case19	−20.13	MALE	5703.52	79	0.098514
Case20	−17.11	MALE	5703.52	77	0.139476
Case21	−17.68	FEMALE	5703.52	77	0.130617
Case22	−26.43	MALE	5899.5	83	0.047698
Case23	−21.73	MALE	5977.89	76	0.081941
Case24	−14.46	MALE	5742.71	71	0.189234
Case25	−20.03	MALE	5938.69	96	0.099655
Case26	−24.56	FEMALE	5860.3	96	0.059156
Case27	−20.22	FEMALE	6213.07	109	0.097499
Case28	−19.32	FEMALE	5703.52	71	0.108143
Case29	−14.9	MALE	4802	75	0.179887
Case30	−19.89	FEMALE	5664.32	106	0.101274
Case31	−25	FEMALE	5742	138	0.056234
Case32	−17	MALE	5233	71	0.141254
Case33	−14.8	MALE	5389	74	0.18197
Case34	−14.9	FEMALE	4802	75	0.179887
Case35	−10.65	MALE	5233.17	93	0.293427
Case36	−12.4	MALE	5193.97	114	0.239883
Case37	−12.18	FEMALE	4762.81	76	0.246037
Case38	−19.56	FEMALE	5625.13	84	0.105196
Case39	−12.23	MALE	5664.32	125	0.244625
Case40	−14.46	MALE	5742.71	71	0.189234
Case41	−14.8	MALE	5389	74	0.18197
Case42	−17	MALE	5233	71	0.141254
Case43	−21.73	FEMALE	5977.89	76	0.081941
Case44	−19.32	FEMALE	5703.52	71	0.108143
Case45	−19.56	MALE	5625.13	84	0.105196
Case46	−26.79	FEMALE	6213.07	72	0.045761
Case47	−26.06	MALE	5936.39	86	0.049774
Case48	−14.8	MALE	5389	74	0.18197
Case49	−14.9	FEMALE	4802	75	0.179887
Case50	−24.56	MALE	5860.3	96	0.059156

**Table 5:** Descriptive statistics.

Parameter	Mean	Std. Deviation	Minimum Value	Maximum Value
S11 (dB)	−21.457800	6.945209	−41.670000	−10.650000
S11 magnitude	0.108798	0.069823	0.008251	0.293427
Frequency (MHz)	5678.402600	372.774235	4762.810000	6252.960000
Invasive Glucose (mg/dL)	84.840000	20.196342	58.000000	163.000000

**Table 6:** Model accuracy summary based on SPSS Modeler [31].

Model	Formula	R^2^/RMSE/MAE (mg/dL)	Visual Performance
Linear Formula	G = 70.3089 + 35.1239·*S*11 + 0.001724·*f*	−0.015/20.14/13.98	Simple trend; high scatter
Polynomial Ridge (degree = 2)	G = −725.78 − 0.01·*S*11 + 0.29574·*f* + 0.77·*S*11^2^ − 0.00125·*S*11·*f* − 0.0000268·*f*^2^	0.053/19.46/13.69	Moderate nonlinearity; improved fit
Random Forest	G = (1/N) $\sum_{i=1}^{N}Ti\left(S11,\;f\right)$ Where Ti stands for tree decision, the dataset was randomly split into 70% training and 30% testing subsets using SPSS Modeler default partitioning	0.721/10.57/5.16	Best fit; lowest error

G = Predicted non-invasive glucose level (mg/dL), *f* is frequency in MHz, S11 is input reflection magnitude, R^2^ is coefficient of determination, RMSE is root mean square error and MAE is mean absolute error.

**Table 7:** State-of-the-art comparison of microwave-based non-invasive glucose sensing approaches with our study.

Ref.	Sensor/Method	Operating Frequency (GHz)	Reported Frequency Shift (MHz)	Design Complexity/Cost	Validation Notes
[32]	Planar resonator (finger-based)	1.8	1.34	Moderate	Non-invasive; limited details
[33]	Dielectric resonator	4.7	0.00281	Complex/costly	Very small shift reported
[34]	Invasive fluid-based sensor	5.41	0.0625	Moderate/low cost	Invasive method
[35]	Wideband microstrip antenna	2.5–18	N/A	Simple/low cost	Shift not reported
[36]	Wearable split-ring resonator	1.5	0.005	Complex	Wearable, low shift
[37]	Interdigitated CPW resonator	2.46	2.0	Complex/costly	Moderate shift
This work	Printed slot antenna with SIR (FR4)	5.7	Sample-dependent	Simple/low cost	50 human-finger samples; ML-based glucose estimation (RMSE ≈ 10.6 mg/dL)

## Data Availability

The original contributions presented in this study are included in the article. Further inquiries can be directed to the author.
